# Parent–Child Relationship Characteristics and Psychiatric Symptoms as Predictors of Passive Suicidal Ideation among Adolescents in Outpatient Substance Use Treatment

**DOI:** 10.1007/s10560-025-01010-4

**Published:** 2025-03-05

**Authors:** Jacquie Lee, Jonathan G. Tubman

**Affiliations:** Department of Psychology, American University, 4400 Massachusetts Avenue NW, Washington, DC 20016 USA

**Keywords:** Adolescents, Substance use, Substance use treatment, Suicidal ideation, Psychiatric disorders, Gender

## Abstract

Adolescents with substance use disorders (SUDs) are at higher risk for negative outcomes including suicidal ideation (SI). This is due in part to high rates of comorbid psychiatric disorders among adolescents diagnosed with a SUD. Among adolescents, SI is a sign of psychological distress that may interfere with substance use treatment engagement and completion. Identification of factors that are significant predictors of SI among adolescents engaged in risky substance use can provide valuable information for customized treatment planning. The present study is a secondary analysis of data from a racially and ethnically diverse sample of 396 adolescents receiving outpatient substance use treatment services to identify predictors of SI. Hierarchical multiple regression analyses were conducted by entering variables sequentially in four blocks: (a) gender, (b) internalizing psychiatric symptoms, externalizing psychiatric symptoms, (c) drug abuse and dependence symptoms, alcohol abuse and dependence symptoms, and (d) negative parent relations, positive parent relations to determine the unique contribution of each block. This analysis revealed that internalizing psychiatric disorder symptoms, gender, and alcohol abuse and dependence symptoms were each significant predictors of mean SI scores. Overall, the results suggested that female clients entering substance abuse treatment may have higher levels of distress than male clients and require more careful screening. Additionally, tailored integrated treatment planning for clients with complex patterns of comorbid internalizing disorder symptoms and alcohol use disorder symptoms may lead to better overall treatment outcomes.

## Introduction

While suicide rates for children and adolescents have plateaued in recent years, suicide, i.e., “the act of intentionally causing your own death” (Evans & Tawk, 2016, p. 55) remains the second most prevalent cause of mortality among persons 10 to 34 years old (Garnett & Curtin, 2023). Adolescence is the developmental period when health risk behaviors associated with suicidal thoughts or behaviors emerge (Wu et al., 2011). Suicidal ideation (SI), conceptualized as “thoughts of engaging in suicide-related behavior” is a persistent public health problem among adolescents in the U.S. (Crosby et al., 2011, p. 90). In 2023, 20% of high school students seriously considered suicide, with SI especially prevalent among females and sexual minority adolescents (Brener et al., 2024).

Researchers tracking the developmental progression of SI and suicidal behaviors note that most people reporting SI do not attempt or complete suicide and that there may be different risk factors for suicidal thoughts vs suicidal behaviors (e.g. Klonsky et al., 2018). However, SI is significantly associated with higher rates of previous suicide attempts and completions, in addition to being a robust sign of substantial psychological distress. Therefore, SI is a critical step in the escalation of suicidal thoughts and behaviors and it indicates an overall lack of psychological health (Jobes & Joiner, 2019; Joiner et al., 2003). Additionally, recent models of suicidal thoughts and behaviors suggest suicidal ideation may escalate from “passive” suicidal ideation—i.e., wishing to be dead—to “active” suicidal ideation—i.e., “I would kill myself if I could”—before escalating to suicidal behaviors, i.e., a suicide attempt (Klonsky et al., 2018). Generally, suicide research combines passive and active suicidal ideation under the umbrella term of “suicidal ideation.” Therefore, research on “passive” suicidal ideation specifically may provide helpful, specific context for what can be the first step in someone’s suicide trajectory. The aim of the present study is to: (1) identify factors that are significant predictors of passive SI in a sample of adolescents receiving outpatient substance use treatment services and (2) describe implications for the delivery of substance use treatment services to adolescents reporting passive SI.

Adolescents are more likely than other age groups to report risky substance use and co-occurring psychological disorders, and adolescents with substance use disorders (SUDs) report some of the highest prevalence rates for SI (Mino et al., 1999; Wilson & Janoff, 2019). Poor family and social relationships are generally associated with increased suicide risk at all ages. Parent–child relationships are especially important for maintaining adolescents’ overall psychological well-being due to the central role family member’s play in negotiating core psychosocial issues of adolescence (Maree, 2021). Pubertal maturation and resulting changes in endocrine processes and brain neural networks can increase suicide risk due to underdeveloped impulsivity control, adaptive stress responses, and emotional regulation during adolescence (Miller & Prinstein, 2019). Changes in neuropsychological processes are partially mediated by changes in peer and family contexts navigated by adolescents (Dahl, 2004). Adolescents are more vulnerable to SI due to stressors associated with multiple (e.g., biological, cognitive, social) co-occurring linked transitions.

Experimentation with alcohol and other drugs is a normative exploratory risk behavior during adolescence (Dawes et al., 2008). In 2023, many adolescents reported past 30-day use of alcohol (22%), marijuana (17%), or prescription opioid misuse (4.4%) (Brener et al., 2024). However, drug experimentation during this period does increase one’s likelihood of chronic drug and alcohol use and subsequent negative cognitive and social consequences (Barnes et al., 2007; Dawes et al., 2008). People in drug treatment who initiated substance use before 18 years old reported worse adult psychosocial functioning, including more psychiatric and behavioral problems, than their treatment peers who initiated substance use at a later age, suggesting early adolescence is an especially risky period to partake in substance use (Poudel & Gautam, 2017). This is likely due to similar biological and cognitive transitions that make adolescents more prone to suicidal ideation (i.e., underdeveloped impulsivity control, weaker emotional regulation and coping skills) along with changes in social relationships and behaviors that are meant to mimic adult life and stoke higher emotional responses or “exhilaration” (Dahl, 2004, p. 21). Additionally, adolescents who experience physical or sexual trauma are three times more likely to report substance use, suggesting alcohol or drugs may be used as coping strategies for adolescents in distress (Khoury et al., 2010).

Alcohol use is consistently associated with increased risk for SI among adolescents (Ellis & Trumpower, 2008; Evans & Tawk, 2016; Wong et al., 2013; Zhang & Wu, 2014). Heavy, episodic alcohol use can increase impulsivity while impairing cognitive functioning, making effective, adaptive problem solving more difficult (Light et al., 2003; Pompili et al., 2010). Long-term heavy alcohol use can also gradually degrade social connections and social support, increasing feelings of isolation and exacerbating depressive thoughts or other psychiatric symptoms that promote the development of SI (Light et al., 2003; Wong et al., 2013; Wu et al., 2004). Similarly, chronic cannabis use during adolescence predicts higher rates of depression, anxiety, and antisocial personality traits among youth (Rasic et al., 2013; Schaefer et al., 2021; Wong et al., 2013). Use of illicit drugs and polysubstance use are also associated with increases in SI (Ellis & Trumpower, 2008; Mino et al., 1999; Rasic et al., 2013; Wong et al., 2013). Overall, the relationship between SUDs and psychological distress may be bidirectional, meaning drug use may increase psychological distress (and subsequent SI risk), or those with psychological distress may use drugs as a coping mechanism (Zhang & Wu, 2014). Identification of significant predictors of SI among adolescents with SUDs is critical because almost 80% of these adolescents are diagnosed with a co-occurring psychiatric disorder, and subsequent SI can interfere with treatment outcomes, either by increasing craving levels or clashing with treatment models that do not address co-occurring psychological issues (i.e. suicidal thoughts and substance use disorders) (Esposito-Smythers et al., 2011; Janakiraman et al., 2020; Wilson & Janoff, 2019).

Psychiatric disorders among children and adolescents are traditionally categorized into either “internalizing” or “externalizing” domains, although they are often comorbid with each other during childhood, while also comorbid with SUDs and SI (O’Neil et al., 2011). Internalizing disorders are psychopathologies with inward-facing emotional and cognitive symptoms, including depression and anxiety; whereas externalizing disorders’ distinct features are outward-facing, behavior-centric symptoms like aggressive or destructive behavior toward oneself or one’s immediate environment (Babicka-Wirkus et al., 2023). Externalizing disorders include conduct disorder, oppositional defiant disorder, and attention-deficit disorders. Internalizing disorders are consistently associated with SI because facets of major depression, such as hopelessness, are integral to SI development (Franklin et al., 2017; Klonsky & May, 2015). Anxiety, and in particular social anxiety, can promote social withdrawal from peers and family members, eroding social support systems, thereby increasing feelings of social isolation and thwarted belongingness, which according to modern models of suicide development are essential components to developing suicidal thoughts (Van Orden et al., 2010). Internalizing disorders, like anxiety or depression, are also associated with low self-esteem and self-efficacy, the lack of which increases vulnerability to developing SI (Symeou & Georgiou, 2017). Externalizing disorders are commonly associated with suicidal behaviors due to elevated agitation or impulsivity, symptoms that promote self-harm, violation of social norms, and subsequent social isolation that comes from social norm violations (Symeou & Georgiou, 2017; Van Orden et al., 2010). Substance use is common in adolescents with both internal and/or external disorders and the relationship between internal and/or externalizing disorders and substance use is likely bidirectional. The prevalence of co-occurring internalizing and externalizing disorders and SUDs is highest during middle to late adolescence, suggesting that late adolescence is a peak period of vulnerability for co-occurring psychiatric problems, SUDs and SI (Wilson & Janoff, 2019).

Parent–child relationships and family cohesion are highly salient for adolescents, making family-level processes a key factor in the development of SI (Hunt et al., 2022). Positive family dynamics may act as a protective factor against SI. Specifically, adolescents reporting higher levels of family support generally have higher self-esteem, which can be an important protective factor against suicidal thoughts and behaviors (Sharaf et al., 2009). However, negative aspects of parent–child relationships such as low acceptance, low emotional sensitivity, and inattentive parenting, are associated with higher risk for SI among adolescents (Wagner et al., 2003; Wang & Qiao, 2022). Low self-esteem or feelings of worthlessness or burdensomeness are core components of models that predict the onset of SI, and suicidal adolescents are more likely to see themselves as burdens to their family compared to non-suicidal adolescents (Klonsky & May, 2015; Van Orden et al., 2010; Wagner et al., 2003). In addition, maladaptive control techniques used during parent–child conflicts are more likely to promote SI development if adolescents see such tactics as invalidating or as impeding their self-direction and autonomy, undermining their self-confidence and self-esteem (Symeou & Georgiou, 2017).

Although suicide research often prioritizes active SI, research on “passive” SI, i.e., suicidal thoughts without a specific plan, is critical because it is an important risk factor for future suicidal behaviors (May et al., 2015). The cumulative prevalence of SI typically increases rapidly during adolescence after remaining low and stable in childhood, resulting in roughly 16% of 17-year-olds reporting SI during their lifetime (Nock et al., 2013). Adolescents in outpatient substance use treatment are more likely to experience additive risk factors for SI, including disrupted family relations and comorbid psychiatric disorders (Hulvershorn et al., 2015; Wong et al., 2013). The goals of the present study were to (a) identify significant predictors of SI among adolescents receiving outpatient substance use treatment services (i.e., psychiatric symptoms, adolescent substance use, and parent–child relationships) and (b) describe potential implications for the delivery of treatment services. We hypothesize that adolescents reporting higher scores for: past-year substance abuse symptoms, past-year internalizing and/or externalizing disorder symptoms, or more difficult parent–child relationship ratings were more likely to report passive SI.

## Methods

### Participants

The present study included a sample of 396 adolescents, including 282 boys and 114 girls receiving substance use treatment services at two outpatient facilities in South Florida. They ranged in age from 12 and 18 years (mean = 16.33 years). Participants identified as Latine (44%), non-Hispanic White (25%), non-Hispanic Black (21%), Asian (5%) or members of other racial or ethnic groups (4%). This study analyzed data from the baseline measurement occasion of a NIAAA-funded (R01 AA014322) evaluation of a brief motivational intervention for HIV-risk reduction among adolescents undergoing outpatient substance use treatment.

### Measures

#### Suicidal Thoughts

Suicidal thoughts were assessed using four questions from the General Health Questionnaire (Goldberg, 1978) that measured recent emotional distress (α = .83). These GHQ items were originally developed to evaluate current levels of psychological distress. The four passive suicidal ideation statements included in this analysis were: the participant “felt that life is not worth living,” “thought about doing away with yourself,” “wishing you were dead and away from it all,” and “the idea of taking your life kept coming into your mind.” Items were answered via a 4-point Likert scale: *Not a Lot* (1), *No More Than Usual* (2), *Rather More Than Usual* (3), or *Much More than Usual* (4). A validity study comparing responses to these four GHQ questions and Beck’s Suicidal Intent Scale indicated these four GHQ questions were appropriate to use as passive SI screeners (Watson et al., 2001).

#### Psychiatric Disorder Symptoms

Past-year psychiatric disorder symptoms were assessed using the Brief Michigan Version of the Composite International Diagnostic Interview (CIDI; Kessler et al., 1998). This measure has been used as a field survey conducted by trained lay interviewers to assess mental disorders in large-scale epidemiological studies. The original CIDI was developed by the World Health Organization (WHO) and has been modified periodically (World Health Organization, 1990). In the present study, the total number of past-year psychiatric symptoms experienced by each participant were aggregated into total scores for each diagnostic category included in analyses. For each symptom, participants responded *Yes* (1) or *No* (0) if they experienced the symptom during the past year. Total symptoms scores were based on DSM-IV diagnostic criteria (American Psychiatric Association, 1994). A higher score for a specific diagnostic category indicated a higher number of past-year symptoms endorsed by participants. Total symptom scores were aggregated for the following disorders: total internalizing symptoms (i.e., total mood disorder and total anxiety disorder symptom scores) and total externalizing symptoms (i.e., total ADHD and total conduct disorder symptom scores), total drug abuse and dependence symptoms and total alcohol abuse and dependence scores. Drug abuse and dependence symptom scores referred to marijuana use, as well as all other forms of illicit substance use (i.e., heroin and cocaine).

### Parent–Child Relations

Parent–child relations were measured via the Children’s Report of Parent Behavior Inventory Short-Form (CRPBI-SF) (Schludermann & Schludermann, 1970). This is an inventory of children’s perceptions of parental behaviors and each item is scored on a 5-point Likert-type scale *Almost Never* (1), *Once in a While* (2), *Sometimes* (3), *A Lot of the Time* (4), or *Almost Always* (5). Given the vulnerable nature of this adolescent sample, we decided to conduct an exploratory factor analysis (EFA) of the items on the CRPBI-SF to determine if the 4 dimensions Schludermann and Schludermann (1970) found in the original SRPBI-SF could be reproduced. Individual items needed to have a minimum factor loading of .40 to load on a specific factor. Items were allowed to load only on a single factor. Using a scree plot and an oblique factor rotation, we identified two underlying dimensions in the CRPBI items for this sample. This EFA was conducted to ensure the CRPBI-SF questions used for this study were appropriate for this vulnerable sample. The first dimension, labeled positive parent–child relations (*n* = 13 items, α = .91), had an eigenvalue of 8.66 and accounted for 22.19% of the variance in the CRPBI items. The second dimension, labeled negative parent–child relations (*n* = 14 items, α = .84) had an eigenvalue of 5.47 and accounted for 14.03% of the variance in the CRPBI items.

### Procedure

Participants were approached during their first week of treatment and were invited to participate in a brief four-session motivational HIV/STI reduction intervention and were subsequently screened for sexual activity within the last 6 months as part of inclusion criteria. Consent from primary caregivers and adolescents was obtained. Clients who were actively suicidal were excluded from study due to IRB-stipulated protections for this vulnerable sample. Clients with significant cognitive impairments were identified by treatment facility and were excluded from the original RCT due to the demands of psychotherapeutic intervention. Structured interviews were conducted by trained graduate research interviewers on an ethnically and racially diverse sample of adolescents. Questions consisted of demographics, past-year psychiatric symptoms, passive suicidal thoughts, risky sexual behaviors, substance use, and family history of substance use. Participants received $25 for participation.

### Analytic Plan

Analyses for the present study were conducted in three steps. First, the distributional characteristics and bivariate correlations among study variables were described for the entire sample. Second, hierarchical multiple regression (HMR) was used for the entire sample to identify significant predictors of total passive SI scores among adolescents receiving outpatient substance use treatment services. In HMR models, predictor variables were entered in blocks to determine the amount of unique variance in SI scores was accounted for by the variables in each block, above and beyond the variance accounted for by previous blocks of variables. Third, a series of one-way analyses of variance (ANOVAs) was conducted to examine differences between participants separated into high and low intensity passive suicidal ideation groups in their mean ratings for past year psychiatric symptoms and parent–child relationship quality. The high severity (*n* = 74) and low severity (*n* = 298) groups were split based on mean passive SI scores (i.e., scores of 1 vs. 2 or higher). This analysis was intended to detect subtle differences between members of higher intensity passive SI and lower intensity passive SI groups that may have been obscured in the HMR models. Analyses were conducted in SPSS Version 28.

## Results

### Descriptive Statistics

Seventy-five percent of participants reported alcohol use sometime in the 180 days before beginning treatment, and most participants reported lifetime substance use, including marijuana (93.6%), opioids (32.0%), hallucinogens (29.0%), amphetamines (19.0%), and heroin (3.1%). Table 1 summarizes the distributional characteristics of study variables for boys and girls in the sample. Girls reported significantly higher mean scores than boys for passive SI (1.31 vs. 1.09, *p* < .01), internalizing disorder symptoms (15.00 vs. 9.40, *p* < .01), and alcohol use disorder symptoms (*M* = 2.19 vs. 1.31, *p* < .01). There were modest, yet significant positive bivariate correlations between (a) passive SI scores and (b) total internalizing disorder symptoms (*r* = .30; *p* < .01); total externalizing disorder symptoms (*r* = .10; *p* < .05), and total alcohol abuse and dependence symptoms (*r* = .27; *p* < .01). While parent–child relationships were not significantly correlated with passive SI, negative parent–child relationship scores were modestly but significantly correlated with total externalizing disorder symptoms (*r* = .16; *p* < .01) and total alcohol abuse and dependence symptoms (*r* = .10; *p* < .05). Conversely, positive parent–child relationships had modest, but significant negative associations with total alcohol abuse and dependence symptoms (*r* = −.13;* p* < .05) total internalizing disorder symptoms (*r* = −.17; *p* < .01), total drug abuse and dependence symptoms (*r* = −.11; *p* < .05), and total alcohol abuse and dependence symptoms (*r* = −.13; *p* < .05). The absolute value of any bivariate correlation (see Table 2) was below .5, indicating multicollinearity was not an issue in subsequent HMR analyses.

**Table 1:** Descriptive statistics for study variables

Variable	*N*	*Mean*	*SD*	Skew	Kurtosis
Females					
Passive suicidal thoughts	114	1.31*	.56	2.22	4.91
Internalizing symptoms	114	15.00*	1.01	.93	.07
Externalizing symptoms	114	19.32	8.25	− .15	− .01
Drug abuse/dependence symptoms	114	4.74	3.83	.20	− 1.35
Alcohol abuse/dependence symptoms	114	2.19*	2.68	1.05	.01
Negative parent relationship	106	35.79	11.11	.46	− .32
Positive parent relationship	106	45.27	13.80	− .24	− 1.11
Males					
Passive suicidal thoughts	282	1.09	.32	5.28	34.87
Internalizing symptoms	282	9.40	6.71	1.91	4.54
Externalizing symptoms	282	18.83	8.01	− .26	− .27
Drug abuse/dependence symptoms	282	4.41	3.37	.18	− 1.22
Alcohol abuse/dependence symptoms	282	1.31	1.97	1.67	2.27
Negative parent relationship	266	37.34	1.58	.32	− .46
Positive parent relationship	266	46.88	11.14	− .65	− .06

Significant mean differences between girls and boys mean scores indicated by * = *p* < .01

**Table 2:** Bivariate correlations among main study variables

Variable	1	2	3	4	5	6	7	8	9
1. Age	–								
2. Gender	− .06	–							
3. Suicidal Ideation Score	− .01	.24**	–						
4. Total Internalizing Symptoms	− .14**	.31**	.30**	–					
5. Total Externalizing Symptoms	− .09	.03	.10*	.41**	–				
6. Drug Abuse/Dependence Symptoms	− .11*	.04	.09	.37**	.36**	–			
7. Alcohol Abuse/Dependence Symptoms	− .05	.18**	.27**	.35**	.30**	.45**	–		
8. Negative Parenting Score	.00	− .06	.08	.09	.16**	.10	.11*	–	
9. Positive Parenting Score	.07	− .06	− .07	− .17**	− .07	− .11*	− .13*	− .25**	–

^*^ = *p* < .05; ** = *p* < .01

### Hierarchical Multiple Regression (HMR) Analyses

The four-step HMR model was implemented with total passive SI scores as the dependent variable. Gender was entered in block one, followed by total internalizing disorder symptom scores and total externalizing disorder symptom scores in block two. Total drug abuse and dependence disorder symptom scores and total alcohol abuse and dependence disorder symptom scores were entered in block three, followed by positive parent–child relationship scores and negative parent–child relationship scores in block four.

The results of the HMR model (see Table 3) revealed that at Step 1, gender contributed significantly to the HMR model *F* (1, 370) = 19.18, *p* < .001 and accounted for 4.9% of the variance in passive SI scores. At Step 2, the inclusion of total scores for internalizing disorder symptoms and externalizing disorder symptoms explained an additional 7.0% of variance in passive SI, and the *ΔR*^*2*^ was significant, *F* (3, 368) = 16.50, *p* < .001. In Step 3, the addition of total scores for drug abuse and dependence symptoms and alcohol abuse and dependence symptoms explained an additional 3.4% of the variance in passive SI, and the *ΔR*^*2*^ was significant, *F* (5, 366) = 13.16, *p* < .001. In Step 4, the inclusion of the positive and negative parent–child relationship scores accounted for only an additional .2% of the variance in Passive SI and this *ΔR*^*2*^ was not significant. The final HMR model was significant, *F*(7, 364) = 9.529, *p* < .001, and accounted for 15.5% of the variance in passive SI. The most important predictors of SI, as indicated by unique additions to the amount of variance accounted for in scores for SI, were total internalizing disorder symptom scores, alcohol abuse and dependence symptoms scores, and gender.

**Table 3:** Full model summary of HMR model predicting suicidal ideation

Variable	β	*t*	sr^2^	*R*	*R*^*2*^	*∆R*^*2*^
Step 1				.222	.049	.049***
Gender	.222	4.380***	.222			
Step 2				.344	.119	.069***
Gender	.137	2.645**	.137			
Internalizing disorder symptoms	.261	4.805***	.243			
Externalizing disorder symptoms	.040	.783	.041			
Step 3				.390	.152	.034***
Gender	.110	2.141*	.111			
Internalizing disorder symptoms	.235	4.226***	.216			
Externalizing disorder symptoms	.013	.257	.013			
Alcohol abuse/dependence symptoms	.212	3.820***	.196			
Drug abuse/dependence symptoms	− .078	− 1.430	− .075			
Step 4				.393	.155	.002
Gender	.115	2.227*	.116			
Internalizing disorder symptoms	.233	4.156***	.213			
Externalizing disorder symptoms	.007	.139	.007			
Alcohol abuse/dependence symptoms	.209	3.757***	.193			
Drug abuse/dependence symptoms	− .080	− 1.452	− .076			
Positive parent–child relations	.008	.150	.008			
Negative parent–child relations	.051	1.012	.053			

* = *p* < .05; ** = *p* < .01; *** = *p* < .001

Following the HMR analyses, we conducted a series of one-way ANOVAs to examine differences in study variables by passive SI group (i.e., high severity, low severity) to generate additional information about possible mechanisms by which these variables influenced passive SI scores. Adolescents in the high severity SI group reported significantly higher mean scores for: total internalizing disorder symptoms *F*(1, 394) = 39.57, *p* < .001 and total alcohol abuse and dependence symptoms *F*(1,394) = 14.04, *p* < .001, as well as significantly lower mean scores for positive parent–child relationships *F*(1,370) = 4.21, *p* < .05. These group differences suggest more subtle differences in relations among psychiatric symptoms, substance use severity, parent–child relationship quality scores and suicidal thought severity that may have been obscured in the initial HMR analyses.

## Discussion

The present study examined substance abuse and dependence symptoms, internalizing and externalizing symptoms, and parent–child relationships as potential predictors of passive SI among adolescents receiving outpatient substance use treatment services. Expected positive associations between substance abuse and dependence symptoms and passive SI were partially supported by our findings, as were expected positive associations between internalizing and externalizing disorder symptoms and passive SI. Consistent with existing literature, participants with higher scores for past-year alcohol abuse and dependence symptoms reported modestly higher mean passive SI scores. However, drug abuse and dependence symptom scores were not significantly associated with passive SI scores. Previous research documents that clinical samples of adolescents tend to report high rates of polysubstance use and that SI is more severe for adolescents who report more substances used (Rodríguez-Arias & Aguilar, 2012; Wong et al., 2013). However, the most commonly used substance in our sample was marijuana, which is not consistently associated with SI (Rasic et al., 2013). The HMR analysis did not distinguish marijuana use alone from polysubstance use or the use of other drugs, which may explain why higher drug use disorder symptom scores was not a significant predictor of passive SI scores.

Participants reporting higher scores for internalizing disorder symptom also reported modestly higher SI scores, consistent with existing literature documenting positive associations between SI and mood disorder and anxiety disorder symptoms (Franklin et al., 2017). This finding suggests that participants entering substance use treatment with a mood or anxiety disorder may be experiencing higher levels of psychological distress compared to their peers. Contrary to our expectations, externalizing disorder symptom scores were not a significant predictor of SI scores. This finding was unexpected based on the typically high rates of externalizing disorder symptoms reported by substance use treatment-seeking adolescents and the high rates of psychiatric comorbidity in clinical samples of adolescents (O’Neil et al., 2011). Externalizing disorder symptoms are primarily behavior-focused, which may explain the lack of a significant association between those symptoms and a cognitively-focused construct such as suicidal ideation. Some research also indicates that externalizing disorder symptoms diminish with age, in particular, after 15 years of age (Wilson & Janoff, 2019). The average age of this sample was 16.33 years, so externalizing symptoms may have been less pronounced and had less impact on psychological distress levels and related SI.

Parent–child relationship scores were not associated with scores for SI in HMR analyses, but subsequent ANOVAs generated some evidence that adolescents in the high intensity SI group reported significantly less positive relationships with their parents. This supports previous research on parent-adolescent relationships and adolescents’ SI, suggesting that positive parent–child relationships are generally related to higher self-esteem among adolescents, which mitigates risk for SI (Sharaf et al., 2009). Family processes are salient to adolescents and relationships with their parents influence how adolescents evaluate their self-worth. Therefore, it was expected that negative parent–child relationship scores would predict scores for SI (Hunt et al., 2022; Plunkett et al., 2007). However, adolescents with negative parent–child relationships may seek other sources of social support outside the scope of our analysis, which may have influenced their self-esteem and subsequent risk for SI.

Female participants reported significantly higher scores for SI, total internalizing disorder symptom scores, and total alcohol abuse and dependence symptom scores, compared to male participants in our study. Clinically significant alcohol use problems and subsequent comorbid psychiatric problems may help explain why female clients in the sample reported higher SI scores than males. Female adolescents tend to report higher levels of alcohol consumption and alcohol use problems than their male peers, and co-morbid mood and anxiety disorders are more common in women engaged in unhealthy alcohol than men (Centers for Disease Control & Prevention, 2023; Jeong et al., 2019). Alcohol use has been shown consistently to moderate the severity of SI (Zhang & Wu, 2014). In addition, females who enter substance use treatment tend to report more traumatic experiences, such as sexual abuse or other forms of maltreatment, compared to their male peers, which are associated with substance use and increased psychological distress and could explain their higher scores for passive SI (Tubman et al., 2021).

### Clinical Considerations and Implications for Social Work Practice

Adolescents diagnosed with SUDs, and girls in particular, should be screened for SI and mood and anxiety disorders upon entering substance use treatment (Jeong et al., 2019). Clients diagnosed with SUDs and other psychiatric disorders often face higher rates of relapse and therefore require tailored substance use treatment to address issues co-occurring with substance use (Hulvershorn et al., 2015). Additionally, social work or health care workers should inquire about adverse childhood experiences (ACEs) or other traumatic experiences at the beginning of treatment due to the strong linkages between SUDs and trauma exposure. Trauma-informed approaches to screening and clinical intervention should be considered to improve management of psychological distress and increase the effectiveness of substance use treatment. The results of our study indicate that specific psychiatric disorder symptoms and alcohol use severity were modestly predictive of passive SI, which suggests that integrated substance use treatment that addresses both clinically significant patterns of substance use and psychiatric disorder symptoms simultaneously is critical.

Integrated substance use treatment ideally should address social determinants of risky substance use, e.g., childhood maltreatment and other adversities, racial or ethnic discrimination or other forms of marginalization, family discord, and community violence. Our findings also suggest that adolescents with internalizing disorders may experience greater psychological distress, potentially increasing their vulnerability to either SI or substance use relapse. When addressing the behavioral health service needs of an adolescent manifesting comorbid internalizing and externalizing disorder symptoms, it may be prudent to triage addressing the internalizing symptoms first. The results of the regression model suggest that addressing dysfunctional parent–child relationship dynamics may be a secondary goal in treatment planning, since results from the ANOVA models suggested that including supportive family members in the treatment process could be beneficial for the recovery process.

The results of the present study support previous research findings that co-occurring psychological issues, and in particular passive SI, are common among adolescents receiving substance use treatment services. A “holistic approach” to substance use treatment is considered best practice by the National Association of Social Workers (NASW, 2013, p. 8). However, variability in standards and quality of social work training for assessing suicide risk and delivery of developmentally appropriate substance use treatment are challenges for health care service delivery (Kourgiantakis et al., 2019). Standardization of training for suicide risk management and addiction treatment, including addiction-specific courses, simulation training and specialized field placements, may facilitate dissemination of holistic treatment models and service delivery (Kourgiantakis et al., 2019). In addition, assessment tools validated in adolescent populations that measure both frequency of substance use and SI, such as the GAIN Short Screener (GSS), would alert providers at the treatment planning stage that a client may need specialized, integrated treatment services (Dennis et al., 2006).

Future research may focus on chronic passive and active SI instead of acute periods of passive SI. Results from this study suggest that having some level of SI continuously may be common among adolescents receiving substance use treatment services. Future research might investigate how chronic SI interacts with motivation for seeking treatment and subsequent treatment outcomes, which would provide social workers potential goals or treatment outcomes to consider when developing treatment plans with adolescents who disclose suicidal ideation. Future studies of adolescents receiving treatment services may also benefit from more robust assessment of SI rather than brief screeners. More holistic assessments of SI would provide more nuanced information about precipitating factors and social or familial contexts related to SI. Additional family processes could also be included in future studies, including a mix of self-reported data and observed behaviors involving multiple informants to improve the ecological validity of studies of clinical samples of adolescents. Social workers may consider including supportive family members in treatment sessions for adolescent clients reporting suicidal thoughts or behaviors. Future research may also include measures of friendship quality or peer relationships given the importance of social influences on adolescent substance use and mental health outcomes. In addition, longitudinal designs, using intensive forms of measurement, could be used to track daily fluctuations in psychiatric symptoms, substance use and SI to better understand their covariation.

### Limitations

A significant limitation of this study was the assessment measure for suicidal ideation, which may have created a floor effect, due to its response formats. Each response option endorsed some level of SI, so it was not possible to know what proportion of participants reported no SI. Poor recall may be a limitation of the self-report data collected in this present study. When clients were screened they were asked to remember instances of substance use or psychiatric symptoms over the past year. Memory loss from intoxication or the passage of time may bias client responses. In addition, self-reports of parent–child relationship quality may be biased against parents who have adolescents with severe behavioral problems or who were court mandated to treatment. The findings of the study, conducted among adolescents receiving outpatient substance use treatment services, may not generalize to adolescents who never received substance use treatment services or who received residential treatment services. Finally, statistical issues related to the non-normality of this sample may have influenced the ability of the regression model to detecting significant predictors of SI.

## Conclusion

The results of this study suggested that internalizing disorder symptoms and alcohol abuse and dependence symptoms were independently associated with the severity of passive SI and may be particularly important influences on the severity of SI risk among adolescents receiving substance use treatment services. In addition, our analyses suggested that girls may enter substance use treatment with more severe substance use or psychological distress than boys. Clinicians treating girls with alcohol use disorder (AUD) symptoms should consider the likelihood of significantly high levels of psychological distress upon entering treatment and tailor treatment planning accordingly.

## Data Availability

Data are available upon request from the first author.
